# Current Clinical Trial Landscape of Gastroenteropancreatic Neuroendocrine Tumors: A New Era of Landmark Trials

**DOI:** 10.3390/jcm14186522

**Published:** 2025-09-17

**Authors:** Supriya Peshin, Shivani Modi, Rodrick Babakhanlou, Junaid Arshad

**Affiliations:** 1 Department of Internal Medicine, Norton Community Hospital, Norton, VA 24273, USA; supriyapeshin720@gmail.com; 2 Jefferson Health, Einstein Healthcare Network, Philadelphia, PA 19141, USA; 3 Division of Hematology and Oncology, University of Arizona, Tucson, AZ 85719, USA; rbabakhanlou@arizona.edu

**Keywords:** GEP-NETs, peptide receptor radionuclide therapy (PRRT), pancreas, gastrointestinal tract

## Abstract

Gastroenteropancreatic neuroendocrine tumors (GEP-NETs) are a heterogeneous group of malignancies characterized by varying degrees of aggressiveness and clinical behavior. Despite advancements in treatment, including targeted therapies such as sunitinib and everolimus, peptide receptor radionuclide therapy with [177Lu]Lu-DOTATATE, and chemotherapy regimens like capecitabine plus temozolomide, the advanced GEP-NETs remain largely incurable. The limited efficacy of current treatments highlights the urgent need for novel therapeutic strategies. Recent years have seen a rise in several landmark clinical trials aimed at exploring new agents and combinations to improve patient outcomes in GEP-NETs. This literature review focuses on the ongoing clinical trials that hold promise for advancing the treatment landscape of GEP-NETs. We include some industry- and cooperative group-sponsored, phase II-III, randomized, and comparative trials in our review. We analyze the design, rationale, objectives, and preliminary findings of these trials, with a particular emphasis on those with pending results that may offer new insights into potential therapeutic targets. By examining these trials, we aim to provide a comprehensive overview of the evolving strategies in the management of GEP-NETs and underscore the importance of continued research innovation in addressing the challenges posed by the heterogeneity of GEP-NETs and in the pursuit of more effective and potentially curative treatment options.

## 1. Introduction

Gastroenteropancreatic neuroendocrine tumors (GEP-NETs) are a diverse and complex group of neoplasms arising from neuroendocrine cells distributed throughout the gastrointestinal (GI) tract [1]. Once considered rare, the incidence of GEP-NETs has steadily increased in recent decades, largely due to advances in diagnostic imaging such as endoscopic ultrasound (EUS), multi-phase computed tomography (CT) and magnetic resonance imaging (MRI) which have enabled earlier and more accurate detection. Additionally, greater clinical awareness and improved disease understanding have contributed to identifying previously undiagnosed cases [2]. While these tumors can develop in any part of the GI tract, including the stomach, small intestine, colon, rectum, appendix, and the pancreas, the small intestine and rectum are the most common sites of origin [3]. The incidence rates for pancreatic and small bowel NETs (sNETs) are 0.7 and 1.2 cases per 100,000 individuals, respectively [4]. Despite their relatively low frequency, GEP-NETs are of growing clinical importance due to their rising prevalence, broad spectrum of clinical presentations, and varied biological behavior, all of which can lead to significant morbidity and can be associated with a significant risk of mortality [5].

A defining feature of GEP-NETs is their clinical and pathological heterogeneity stemming from the ability of neuroendocrine cells to synthesize and secrete a wide range of hormones and biologically active peptides. Based on this secretory function, GEP-NETs are broadly classified into functional and non-functional types [6]. Functional tumors produce hormones that lead to distinct clinical syndromes, accounting for approximately 10–30% of pancreatic neuroendocrine tumors (pNETs), whereas the more common non-functional tumors, comprising about 70–90% of cases, are often asymptomatic and discovered incidentally or due to mass effect or metastasis [7].

**Figure 1:**
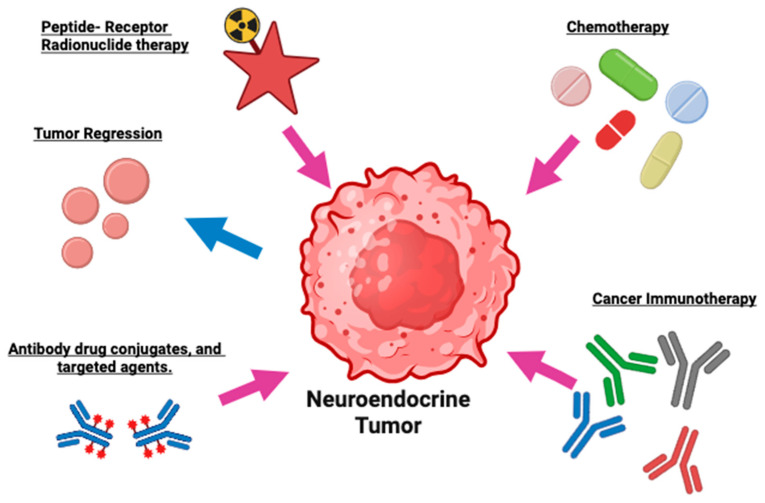
Schematic illustration of therapeutic approaches for neuroendocrine tumors (NETs). This figure illustrates a central neuroendocrine tumor cell being targeted by five major therapeutic strategies. Peptide-Receptor Radionuclide Therapy (PRRT) involves delivering targeted radiation to tumor cells using somatostatin receptor-binding peptides conjugated with radionuclides, thereby enabling precise tumor cell ablation. Chemotherapy employs cytotoxic drugs to interfere with tumor cell division and promote apoptosis, contributing to tumor shrinkage. Cancer immunotherapy utilizes immune-modulating agents such as monoclonal antibodies to activate and direct the host immune system against NET cells. Antibody-drug conjugates and targeted agents merge highly specific antibodies with cytotoxic compounds to deliver lethal payloads directly to cancer cells, minimizing collateral damage to normal tissue. Finally, tumor regression symbolizes the cumulative therapeutic effect of these interventions, manifesting as a reduction in tumor size and disease burden.

The disease spectrum ranges from slow-growing indolent tumors to highly aggressive forms, posing challenges for diagnosis, classification, and treatment selection [1,4,5]. An overview of established and investigational treatment strategies is presented in Figure 1, highlighting current therapeutic options such as peptide receptor radionuclide therapy (PRRT), chemotherapy, immunotherapy, antibody drug conjugates, and targeted agents [8].

## 2. Pathophysiology

The pathophysiology of GEP-NETs is highly complex and not yet fully understood, involving a multifaceted interplay of genetic, epigenetic, and molecular alterations. At genetic level, mutations in key regulatory genes, including Multiple Endocrine Neoplasia Type 1 (MEN1), Death Domain-Associated Protein 6 (DAXX), Alpha Thalassemia/Intellectual Disability Syndrome X-Linked (ATRX), and those affecting the mammalian target of the rapamycin (mTOR) signaling pathway, disrupt normal cellular mechanisms such as proliferation and apoptosis in pancreatic neuroendocrine tumors (pNETs) [6]. For instance, mutations in MEN1 alter chromatin remodeling and transcriptional regulation, while mutations in DAXX and ATRX affect chromatin stability and telomere function, contributing to tumorigenesis. Dysregulation of critical signaling pathways, such as the Phosphatidylinositol 3-Kinase (PI3K)/AKT Serine/Threonine Kinase (AKT)/mTOR and Wnt/β-catenin pathways, further promotes uncontrolled tumor cell growth, survival, and metastasis [6]. The molecular landscape of small bowel neuroendocrine (sbNETs) is characterized by a relatively low mutation rate compared to other cancers and usually includes copy number alterations such as frequent loss of chromosome 18, epigenetic alteration, and cyclin dependent kinase1B (CDKN1B) alterations [9]. These alterations highlight the molecular complexity of GEP-NETs and provide opportunities for targeted therapeutic interventions. NETs are also particularly notable for their pronounced vascularity. Specifically, GEP-NETs exhibit an intratumoral density of blood vessels that is approximately 10-fold higher compared to many other carcinomas. This elevated vascular density plays a crucial role in influencing their biological behavior and therapeutic selection. The rich vascular network is thought to facilitate nutrient delivery and potentially impact the tumor’s growth dynamics and metastatic potential [7].

The tumor microenvironment also plays a pivotal role in supporting GEP-NET growth and metastasis. Angiogenesis, driven by vascular endothelial growth factor (VEGF) and other proangiogenic factors like Fibroblast Growth Factor (FGF), Platelet-Derived Growth Factor (PDGF), and Transforming Growth Factor-Beta (TGF-β), is in particular crucial for tumor progression, especially in metastases to highly vascularized organs such as the liver [8]. This proangiogenic milieu not only supplies nutrients and oxygen to the tumor cells but also facilitates the dissemination to distant sites. Additionally, the overexpression of somatostatin receptor subtype 2 (SSTR2) is considered a hallmark of many GEP-NETs. SSTR2 plays a central role in both tumor biology and clinical management. It mediates the inhibitory effects of somatostatin on hormone secretion and cell proliferation, while its overexpression underpins current imaging techniques and targeted therapies such as somatostatin analogs and radioligand treatment [10,11]. Its exact role in tumor progression remains complex, potentially involving modulation of tumor cell signaling and interactions within the tumor microenvironment [12]. Together, these genetic, molecular, and microenvironmental mechanisms highlight the biological complexity of GEP-NETs and continue to shape the development of precision-targeted therapies.

## 3. Classification

The World Health Organization (WHO) classifies NETs based on histologic differentiation and proliferative index, measured by mitotic count and Ki-67 labeling [13]. This framework divides NETs into three grades: low-grade (G1, Ki-67 < 3%), intermediate-grade (G2, Ki-67 3–10%), and high-grade (G3, Ki-67 > 20%), reflecting the tumor’s aggressiveness and prognosis [13]. This grading is essential for guiding treatment decisions and prognostication. GEP-NETs are further categorized as functional or non-functional based on hormone production [14]. Functional tumors secrete bioactive substances such as insulin, glucagon, vasoactive intestinal peptide (VIP), and gastrin in cases of pancreatic NETs (pNETs) or 5-hydroxyindoleacetic acid (5-HIAA) in cases of small bowel NETs, which can lead to clinical syndromes, such as the carcinoid syndrome [8]. Non-functional tumors do not produce hormonally active substances but may present with symptoms related to tumor bulkiness or metastatic spread [15].

## 4. Clinical Presentation

The clinical presentation of GEP-NETs varies significantly between localized and metastatic disease [16]. In the localized setting, patients may be completely asymptomatic, with tumors often discovered incidentally during routine imaging or endoscopic procedures. When symptoms are present, they are typically nonspecific—and can include abdominal pain, mild gastrointestinal disturbances, or subtle changes in bowel habits. Functional tumors, even when localized, can present with distinct clinical syndromes based on their hormone secretion patterns: for instance, insulinomas may cause hypoglycemic episodes, gastrinomas can result in severe peptic ulcer disease (Zollinger–Ellison syndrome), and VIPomas may lead to severe diarrhea and electrolyte imbalances [17]. The symptoms play an important role in the initial diagnosis.

At the time of diagnosis, nearly 50% of GEP-NETs are already metastatic, and symptomatology becomes broader and more severe in this context [18,19]. Common manifestations include significant weight loss, fatigue, and worsening abdominal symptoms. Liver metastases are particularly impactful, often leading to carcinoid syndrome, which is characterized by episodic flushing, diarrhea, wheezing, and potential right-sided heart valve problems due to the systemic effects of vasoactive substances such as 5-HIAA. Organ-specific symptoms may also occur—such as hepatomegaly and right upper quadrant pain (liver involvement), bone pain (skeletal metastases), and lymphadenopathy (lymph node spread). Early detection and accurate staging are critical, as localized tumors may be curatively resected, whereas metastatic disease typically requires a multidisciplinary decision-making approach with a focus on systemic therapy and often associated with a poor prognosis [19].

## 5. Treatment

The treatment landscape for GEP-NETs encompasses various therapeutic approaches tailored to tumor type, grade, stage, and functionality. Surgical resection remains the cornerstone of treatment for localized disease and offers the only curative potential. For pNETs the 5-year overall survival (OS) following complete (R0) resection range from 60 to 75%, with higher-grade tumors exhibiting poorer outcomes. In contrast, sNETs typically demonstrate more indolent behavior, with 5-year OS rates of 80–100% after completing resection [20].

In patients with small (<20 mm) non-functional PanNETs, observation may be appropriate, especially in tumors with intact ATRX and DAXX genes. Well-differentiated pNETs frequently harbor mutations in ATRX and DAXX genes involved in chromatin remodeling and telomere maintenance.

Loss of ATRX/DAXX expression correlates with shorter disease-free and OS, indicating a more aggressive clinical course despite well-differentiated histology, even though this prognostic value may vary depending on stage [21].

Metastatic tumors which make almost half of the newly diagnosed GEP-NETs require systemic treatment. Somatostatin analogs (SSAs) are typically used as first-line therapy in functional and non-functional NETs that are well differentiated and express somatostatin receptors, typically in G1/G2 tumors (Ki-67 ≤ 10%). SSAs can also be used in combination regimens for more aggressive tumors.

For metastatic pNETs, median OS ranges from 24 to 60 months, with more recent studies reporting extended survival up to 42–60 months with current treatment modalities [22]. Metastatic sNETs exhibit a more favorable prognosis, with median OS ranging from 56 to 104 months [23,24]. This distinction highlights the biological heterogeneity of GEP-NETs and the need for site-specific treatment approaches [25].

The PROMID and CLARINET studies are two landmark clinical trials which confirmed the antiproliferative efficacy of SSAs. The PROMID trial showed that octreotide LAR prolonged median progression free survival (mPFS) to 14.3 months versus 6 months with placebo, with a disease control rate (DCR) of 66.7%, and an objective response rate (ORR) of 2.4%. The hazard ratio (HR) was 0.34 (95% CI: 0.20–0.59; *p* < 0.001) [26]. Similarly, the CLARINET evaluated lanreotide in non-functional enteropancreatic NETs, reporting mPFS of 32.8 months versus 18 months for placebo. At 24-month PFS rate of 65.1% in the lantreotide group versus 33% for placebo, with a DCR of 87%, and ORR of 2.3%; HR was 0.47 (95% CI: 0.30–0.73; *p* < 0.001) [27].

Targeted therapies including the mTOR inhibitor everolimus, have also become integral. In the RADIANT-3 trial, everolimus, extended mPFS to 11.0 months versus 4.6 months with placebo in patients with metastatic G1 and G2 pNETs after initial progression on SSAs. This study also showed an ORR of 5%, a DCR of 78%, and an HR of 0.35 (95% CI: 0.27–0.45; *p* < 0.001) [28]. Radiant 4 trial showed the efficacy of everolimus in the G1 and G2 advanced sNETs. This trial showed a mPFs of 11.0 months as compared to 3.9 months [29].

Chemotherapy, including capecitabine plus temozolomide (CAPTEM), plays an important role in metastatic pNETs especially in patients with heavy liver involvement, bulky tumors, high disease burden or functional tumors requiring a faster treatment response [30]. Frontline peptide receptor radionuclide therapy (PRRT) is generally reserved for well differentiated, SSRT-positive tumors, especially in patients with Ki-67 ≥ 10% [31] or in subsequent lines after failure of SSAs [8].

Therapy selection is guided by tumor grade and differentiation, growth rate, somatostatin receptor expression, tumor burden and location, prior treatments, performance status, comorbidities, and patient preferences [32]. Despite advances, treatment remains largely non-curative in metastatic disease, aiming instead to extend PFS and preserve quality of life.

Key challenges include the lack of validated biomarkers, optimal sequencing strategies, and definitive data to guide treatment for functional versus non-functional NETs, particularly in metastatic settings [33,34,35,36]. Ongoing federally funded and industry sponsored trails are investigating novel targeted agents, immunotherapies, and rational combinations that may redefine treatment paradigms [37].

Biomarkers are essential tools in the clinical management of NETs, including GEP-NETs, where they support diagnosis, prognostication, and therapeutic monitoring. Among traditional biomarkers, Chromogranin A (CgA) is the most commonly used, which can be elevated in 80–90% of cases. However, its specificity is affected by proton pump inhibitors, renal dysfunction, and non-malignant conditions, and has constrained its clinical utility [38,39]. To overcome these limitations, multianalyte molecular assays such as the NETest have been developed. NETest is a blood-based assay that evaluates circulating transcriptomatic signatures and demonstrates a diagnostic accuracy of ~91%, outperforming CgA. It correlates with tumor burden, grade, and imaging findings, offering dynamic insight into disease progression and treatment response [40]. Emerging data suggests its utility in surgical decision-making and early recurrence detection, pending further evaluation [41]. Other investigational biomarkers include circulating tumor cells (CTCs) and microRNAs, which show promise as minimally invasive tools, though standardization and clinical validation remain ongoing [42,43]. The integration of novel molecular biomarkers into clinical practice has the potential to significantly enhance personalized management strategies in patients with NETs and has been the subject of several ongoing clinical trials.

This article provides a comprehensive narrative review of the evolving GEP-NET treatment landscape, summarizing major ongoing clinical trials, addressing critical knowledge gaps, and highlighting opportunities to improve outcomes through precision oncology and evidence-based therapeutic sequencing [8].

## 6. Targeted Treatment in GEP-NETs

### 6.1. Radioligand Treatment/Peptide Receptor Radionuclide Therapy (PRRT)

PRRT is a targeted molecular approach that utilizes radiolabeled somatostatin analogs typically linked to a radionuclide like lutetium-177 delivered via a chelator to bind SSTR2 on neuroendocrine tumor cells. Once bound, the complex is internalized, delivering cytotoxic radiation to the tumor cell nucleus. PRRT has shown substantial benefit in patients with advanced GEP-NETs, particularly those with strong SSTR expression [44].

(177Lu)Lu-DOTATATE is a beta emitting PRRT with a longer path length and low linear energy transfer. The pivotal phase III NETTER-1 trial evaluated (177Lu)Lu-DOTATATE in patients with progressive midgut NETs and demonstrated a mPFS of 28.4 months compared to 8.5 months with high-dose octreotide, an ORR of 18%, DCT of 81%, and HR for progression or death of 0.21 (95% CI: 0.13–0.33; *p* < 0.001) [44,45].

The ongoing NETTER-2 trial expands on this by evaluating (177Lu)Lu-DOTA-TATE in newly diagnosed, higher-grade (G2 with Ki-67 >10% and G3 ≤ 55%) GEP-NETs, including pNETs and sNETs. Preliminary results published in 2024 indicate the trial met its primary endpoint of improved PFS 22.8 months as compared to 8.5 months with a notable ORR of 43%. Secondary outcomes such as quality of life and overall survival are still under evaluation [31].

An important long-term consideration with PRRT is the risk of myelodysplastic syndrome (MDS), with an estimated incidence of 2–3%, especially in patients previously exposed to alkylating agents or external beam radiation. Regular hematologic monitoring is recommended during and after treatment [46]. A summary of key ongoing and completed trails evaluating radioligand and targeted therapies is provided in Table 1.

### 6.2. Novel Radioligand Agents

(225Ac)DOTATATE (RYZ101):

Alpha-emitting radionuclides like Actinium-225 (225Ac) offer high cytotoxic potency and short tissue penetration, making them ideal for targeting micro-metastatic disease. In a prospective study of 32 patients with [177Lu]Lu-DOTATATE-refractory, SSTR-positive GEP-NETs, (225Ac)DOTATATE achieved a partial response in 15 patients and stable disease in 9 patients, with no documented disease progression [47]. This agent is currently under investigation in the ACTION-1 trial (NCT05477576), which is a phase Ib/III study comparing RYZ101 to standard-of-care therapies in patients with SSTR2 positive advanced GEP-NETs refractory to (177Lu)Lu-DOTATATE therapy [48].

(212Pb)DOTAMTATE:

(212Pb)DOTAMTATE is also a form of targeted alpha PRRT, a bifunctional metal chelator [DOTAM] and the SSTR-targeting peptide [TATE]. A phase I dose escalation study showed the safety and tolerability in patients with advanced refractory disease setting. In the Phase I trial, five out of eight PRRT- naïve subjects with SSTR+ GEP-NETs treated with the same regimen of ^212^Pb-DOTAMTATE achieved an ORR 62.5%. ALPHAMEDIX 02 is a Phase II, open-label, multicenter study evaluating the safety, tolerability and efficacy of ^212^Pb-DOTAMTATE in PRRT-naïve (Cohort 1, N = 36) and PRRT-refractory (Cohort 2, Target N = 30). The results from cohort 1 show an ORR of 47.2% in the treatment naïve patients, which is higher than the results reported in the NETTER-1 study. The final results from this study are pending (NCT05153772) [49].

### 6.3. Comparative Trials

Due to several different novel treatment targets, the treatment sequencing remains an answered question. There are clinical trials underway to determine the comparative efficacy of different agents.

The Canadian Cancer Trials Group (CCTG) in collaboration with Southwestern Oncology Group (SWOG) is running the NETRetreat trial. This phase II trial compares the effect of retreatment with two additional cycles of (177)Lu-DOTATATE PRRT to the usual approach of treatment with everolimus in patients who have previously received (177)Lu-DOTATATE for metastatic and unresectable midgut neuroendocrine tumors. The trial excludes patients who have received prior chemotherapy with capecitabine and temozolomide to avoid the long-term risk of myelodysplastic syndrome due to radiation toxicity. Currently this trial is actively enrolling, and no preliminary results are available. The results from this trial will answer the next best treatment option after initial treatment with (177)Lu-DOTATATE (NCT05773274) [50]. With the availability of novel radioligand agents with alpha particles, the role of Lutetium dotatate after initial progression remains yet to be explored.

The efficacy of capecitabine plus temozolomide is directly compared to Lu-177 Dotatate in advanced or metastatic *pNET* in ComPareNET trial. Currently both agents are available in the frontline treatment of advanced/metastatic pNETs with comparable efficacies and toxicities, however both the agents have not been directly compared in a randomized clinical trial setting. This trial is led by Alliance for Clinical Trials in Oncology and is available at most neuroendocrine centers across the United States. The primary end point is progression free survival (PFS), and the secondary end points include ORR, duration of response (DOR), time to tumor progression and quality of life (QoL). No preliminary results are available (NCT01824875) [30].

COMPETE is a prospective, randomized, controlled, open label, phase III study to evaluate the efficacy and safety of Lu-177 Edotreotide in comparison to everolimus in patients with advanced/metastatic SSTR(+) GEP-NETs. This is an international trial including 200 patients in the Lu-177 Edotreotide arm and 100 in the everolimus arm. This trial uses edotreotide, an octreotide analog with similar efficacy and toxicity. The primary endpoint is mPFS, and the secondary endpoints include the ORR, partial or complete response (PR/CR) and duration of disease control. Some of the other secondary study end points include safety, tolerability, dosimetry measures, overall survival (OS), and QoL. Preliminary results show a mPFS of 23.9 months as compared to 14.1 months. Final results are pending (NCT03049189) [51].

COMPOSE trial is a prospective, randomized, controlled, open label, multi-center, phase III study in patients with well-differentiated high grade 2 and grade 3 (Ki-67 index 15–55%), SSTR (+) GEP-NETs [43]. This trial will evaluate the efficacy, safety, and patient reported outcomes of first- or second-line treatment with Lu-177 Edotreotide compared to the best standard of care. The study plans to randomize 202 patients in 1:1 to either RLT or an active comparator arm. The comparator arm includes either chemotherapy with capecitabine plus temozolomide or 5-fluorouracil plus oxaliplatin or everolimus as per the investigator’s choice. The primary endpoint is PFS, and the secondary end points include OS. The study recruitment commenced in September 2021, and is currently ongoing with no preliminary results (NCT04919226) [52].

### 6.4. Radioligand Combination Trials

(177)Lu-DOTATATE has shown an ORR of 44% in the front-line treatment of advanced GEP-NETs. In order to improve the ORR, several novel agents are being used in combination with RLT.

- DNA Repair Enzyme Inhibitors: Combining (177)Lu-DOTATATE with DNA repair enzyme inhibitors enhances the therapeutic effect by increasing DNA damage in neuroendocrine tumor cells [44]. This synergy results in more effective tumor cell kill and potentially improved patient outcomes. These inhibitors prevent the repair of radiation-induced DNA breaks, amplifying (177)Lu-DOTATATE efficacy in treating neuroendocrine tumors. Peposertib is a deoxyribonucleic acid protein kinase (DNA-PK) inhibitor which can enhance the DNA damage caused by (177)Lu-DOTATATE. DNA-PK is a DNA repair enzyme which when upregulated enhances repair of double stranded DNA breaks causing resistance to radiation induced DNA damage. The combination of peposertib and (177)Lu-DOTATATE is currently being studied in an investigator initiated, multi-center phase I clinical trial in patients with well differentiated GEP-NETs after failure of at least one prior line of systemic treatment. Patients with prior RLT will be excluded. The study plans to enroll 29 patients. No current results are available [53].

Triapine is a ribonucleotide reductase (RNR) inhibitor which is an enzyme involved in the conversion of ribonucleoside diphosphate to deoxyribonucleotide diphosphate, the key building block for DNA synthesis. Combining Lu-177 Dotatate with triapine will enhance the double stranded DNA breaks leading to better efficacy. This novel combination is currently being studied in an investigator initiated, multi-center phase one clinical trial in patients with well differentiated GEP-NETs after failure of at least one prior line of systemic treatment. Patients with prior RLT will be excluded. The study plans to enroll 29 patients. The results from the phase I study are not published. Currently a phase II trial is underway to confirm the safety and therapeutic benefit (NCT04234568) [54].

Olaparib is a selective and potent inhibitor of poly(adenosine diphosphate-ribose) polymerase (PARP) enzymes, PARP-1 and PARP-2 causing the inhibition of DNA repair mechanisms. Combining with (177)Lu-Dotatate will lead to possible synergism with double stranded DNA breaks with radiation leading to increased potential efficacy in patients with advanced/inoperable GEP-NETs. Currently, this combination is under investigation in a National Cancer Institute (NCI) sponsored clinical trial, with no preliminary results (NCT04086485) [55].

II. Immune Check Point Inhibitors: Immune check point inhibitors (ICIs) work by blocking the interaction of program death (PD) proteins and program death ligands 1 (PD-L1) expressed on immune and cancer cells, respectively, thus removing the inhibition of the immune system to kill cancer cells. Limited animal study data is available for the combination of PRRT and ICIs citing no safety concerns. A phase I study showed safety and favorable toxicity in lung NETs. Currently, a phase II trial evaluates the combination of 177-Lu DOTATE with nivolumab (anti-PD1) in adult patients with grade 3 advanced neuroendocrine tumors and neuroendocrine carcinomas. The trial is ongoing, and no results are available (NCT04525638) [56].

**Table 1:** Current clinical trial landscape of gastroenteropancreatic neuroendocrine neoplasms.

Trial ID	Drug	Cancer Type	Phase	Status
NCT05477576	[225Ac]DOTATATE (RYZ101) [47]	Advanced, well-differentiated GEP-NETs	III	Active
NCT05153772	[212Pb]DOTAMTATE [49]	unresectable or metastatic somatostatin receptor-expressing GEP-NETs	II	Active
NCT05773274	Lutetium (Lu-177) Dotatate [50]	Advanced, well-differentiated GEP-NETs	II	Active
NCT03049189	Lu-Edotreotide [51]	SSTR+- G1/G2 advanced/metastatic GEP-NETs	III	Active
NCT04919226	Lu-Edotreotide [52]	G2/G3 advanced/metastaticGEP-NETs	III	Active
NCT05724108	triapine plus [177]Lu DOTATATE [54]	SSTR-GEP-NETs	II	Active
NCT04086485	Olaparib [55]	GEP-NETs	I/II	Active
NCT04525638	Nivolumab [56]	NET and Neuroendocrine carcinomas	II	Unknown
NCT06041516	Antibody Drug conjugate ADCT-701 [57]	NET and Neuroendocrine carcinomas	I	Active
NCT06943755	Zanzalintinib	Advanced or Metastatic NETs	II/III	Actice
NCT05040360	Capecitabine and Temozolomide	High-risk pNET	II	Active

Neuroendocrine Neoplasms—NENs, Mechanism—Mech., Therapeutic Target—Target, Combination Therapy—Combo Tx, Cancer Type—CA Type, Phase—Ph, Progression-Free Survival—PFS, Overall Survival—OS, Serious Adverse Events—AE, Radioligand Therapy—RLT, Somatostatin Receptor 2—SSR2, Lutetium (Lu-177) Dotatate—Lu-177 Dotatate, Metastatic Gastroenteropancreatic, Neuroendocrine Tumors—GEP-NETs, FDA Approved—FDA Appr., Neuroendocrine Tumors—NETs.

## 7. Tyrosine Kinase Inhibitors (TKIs)

Due to increased vascularity of GEP-NETs, several TKIs have been studied in the management of GEP-NETs but only two TKIs have been approved by Food and Drug Administration (FDA) so far. Sunitinib is a multityrosine kinase inhibitor approved in the treatment of metastatic pNETs after initial progression on SSAs based on SUN111 trial. Results demonstrated that sunitinib improved mPFS (11.4 months versus 5.5 months; HR ~0.42; *p* < 0.001) and achieved an ORR of 9.3%. This trial was terminated early due to safety and efficacy issues. Although OS favored sunitinib the difference did not reach statistical significance likely confounded by the crossover from the placebo to interventional arm. The side effect profile was consistent with VEGF class effect [58].

Cabozantinib is a multi-tyrosine kinase inhibitor evaluated in randomized phase III CABINET trial in advanced pNETs and extrapancreatic NETs (epNETs) after progression on two lines of treatment. The results demonstrated a mPFS of 13.8 months versus 4.4 months in pNETs (HR 0.23, 95% CI: 0.12–0.42; *p* < 0.001) and 8.4 months versus 3.9 months in extra-pancreatic NET (HR 0.38, 95% CI: 0.25–0.59; *p* < 0.001), leading to FDA approval in both settings [59,60]. Cabozantinib is the only TKI approved in the treatment of advanced progressive small bowel neuroendocrine tumors. The ORR of cabozantinib in pNETs is double (18%) the ORR of sunitinib, attributed to the widespread inhibition of other targets such MET, AXL, etc. Patient-reported outcomes, including assessments from validated instruments like European Organization of Research and Treatment of Cancers (EORTC) Quality of Life (QoL) Questionnaire-C30, showed a greater incidence of treatment-related symptoms notably fatigue, diarrhea, decreased appetite, and palmar-plantar erythrodysesthesia in the cabozantinib arm compared to placebo. These side effects had the potential to negatively affect physical functioning and daily activities. However, despite the increase in symptomatic toxicity, many patients remained on therapy due to the clinical benefit in disease control, and the side effects were generally manageable with dose modifications and supportive care. Importantly, the delay in disease progression provided by cabozantinib may translate into a longer period of symptom stability, which can support preservation of overall quality of life in patients with progressive, treatment-refractory pNETs. Thus, while cabozantinib introduces toxicity-related challenges, it may offer a meaningful tradeoff between disease control and QoL, particularly in patients with limited therapeutic options [61].

Zanzalintinib (XL092) is a next-generation, oral multi-targeted TKI that inhibits VEGFR, MET, AXL, and MER kinases. It is currently being evaluated in multiple tumor types, including GEP-NETs, for its potential to block tumor angiogenesis and growth while minimizing off-target toxicity. STELLAR-311 is a planned global, randomized, open-label Phase III pivotal trial evaluating zanzalintinib (XL092) versus everolimus as a firstline oral therapy in patients with advanced neuroendocrine tumors (NETs), regardless of site of origin (including gastroenteropancreatic NETs) (NCT06943755). The trial will be starting in the near future.

## 8. Chemotherapy

The ECOG-ACRIN E2211 trial was a multicenter, randomized phase II study conducted in patients with advanced, low- or intermediate-grade pNETs who had documented progression within the prior 12 months. A total of 144 patients (72 per arm) received either temozolomide monotherapy or capecitabine plus temozolomide (CAPTEM); the primary endpoint was progression-free survival (PFS), with secondary endpoints including overall survival (OS), objective response rate (ORR), safety, and exploratory evaluation of MGMT status by immunohistochemistry and promoter methylation. At interim analysis, median PFS was significantly longer with CAPTEM at 22.7 months versus 14.4 months (HR = 0.58; *p* = 0.022), meeting the primary endpoint. Final analysis showed a non-statistically significant but clinically meaningful OS benefit (median 58.7 vs. 53.8 months; HR = 0.82; *p* = 0.42). ORRs were robust at 40% with CAPTEM and 34% with temozolomide alone, and MGMT deficiency was associated with higher response rates. This chemotherapy combination is commonly used in clinical practice; however, has not been approved by FDA [30]. 

The S2104 trial (NCT05040360) is a randomized phase II study comparing CAPTEM versus observation in patients with resected, well-differentiated grade 2 or 3 pNETs (Ki-67 ≥ 3%–≤55%) and a Zaidi score ≥ 3. Patients who underwent resection or ablation of up to five liver metastases were also included, provided they achieved no evidence of disease before enrollment. The trial aims to assess the potential role of CAPTEM in the adjuvant setting. The primary end point is the recurrence free survival and the secondary end points include the overall survival, with safety. Currently, no results are available [62].

## 9. Miscellaneous

ADCT-701 is a novel pyrrolobenzodiazepine (PBD) dimer–based antibody drug conjugate (ADC) developed by ADC Therapeutics, consisting of a humanized IgG1 antibody directed against Delta-like 1 homolog (DLK1). DLK1 is a transmembrane protein involved in developmental signaling that is re-expressed in certain neuroendocrine tumors. Its overexpression has been associated with tumor proliferation, stemness, and poor prognosis, making it a potential biomarker and therapeutic target, particularly in high-grade and aggressive NETs. The agent is currently undergoing a first-in-human Phase I dose-escalation trial (NCT06041516) in adults with advanced neuroendocrine neoplasms (NETs and NECs) or adrenocortical carcinoma, using a classic 3 + 3 design across up to 10 dose levels, enrolling up to 70 evaluable patients to establish the maximum tolerated dose (MTD) and recommended phase II dose. The primary endpoint is dose-limiting toxicity/safety, with secondary and exploratory measures including preliminary anti-tumor activity by response evaluation in solid tumors (RECIST) criteria, pharmacokinetics, response rate, and overall survival [57].

PEN-221 is a miniaturized somatostatin receptor 2 (SSTR2)-targeted antibody-drug conjugate that links an octreotate agonist to the cytotoxic payload DM1 being evaluated in a phase I/II clinical trial. In the dose-escalation phase I portion, the maximum tolerated dose (MTD) was established initially 18 mg every 3 weeks and later amended to a body surface area-based dose of 8.8 mg/m^2^. The expansion cohort enrolled 32 patients with advanced, well-differentiated, SSTR2-positive GI mid-gut or pancreatic NETs who had disease progression [63]. The primary endpoints were safety and tolerability leading to recommended dose for Phase II, and in the expansion cohort, clinical benefit rate (CBR) per RECIST 1.1 along with mPFS as secondary measures; exploratory endpoints included objective response rate (ORR), duration of response (DOR), pharmacokinetics, and immunogenicity (anti-PEN-221 antibodies). PEN-221 demonstrated efficacy at 8.8 mg/m^2^ with a CBR of 88.5% and mPFS of 9 months. Of the 26 patients who were evaluable for response, 23 (88.5%) had stable disease (SD) reported as their best response and target lesion shrinkage was observed in 10 (38%) patients [64].

## 10. Discussion/Future Directions

The evolving therapeutic structure of GEP-NETs reflects significant strides in precision oncology. Emerging modalities such as radioligand therapies, tyrosine kinase inhibitors, immune checkpoint inhibitors, and antibody–drug conjugates are transforming management paradigms by offering tumor-selective mechanisms that expand beyond traditional cytotoxic approaches [65,66]. Landmark clinical trials continue to define long-term safety, efficacy, and integration of these therapies into real-world treatment sequences, which remain an active area of investigation.

Despite these advancements, several key challenges persist. One major hurdle is the heterogeneity of these tumors, even among patients with similar clinical profiles. Additionally, while many of the novel agents show promising response rates and PFS data, they are frequently accompanied by adverse events that may impair quality of life especially in patients with indolent diseases. These adverse events add significant morbidity, adding further complications in the management of these tumors. High treatment costs and accessibility of these advanced treatments also limit the widespread implementation of these therapies. Furthermore, resistance mechanisms and data on long-term disease control remain underexplored, underscoring the need for ongoing translational research and refinement of combination regimens [67].

The most optimal way to handle these challenges includes a multidisciplinary approach. The multidisciplinary management of advanced GEP-NETs requires a patient-centered, biologically informed approach that integrates expertise from medical oncology, surgery, nuclear medicine, endocrinology, and radiology. Key patient-related factors include age, performance status, comorbidities, and symptom burden which must be carefully balanced with tumor-specific characteristics, including tumor grade, differentiation, disease burden, metastatic distribution, and somatostatin receptor expression. Equally critical is the underlying tumor biology, including Ki-67 index, functional status, and emerging molecular markers, which guide therapy selection from options like somatostatin analogs, targeted therapies, PRRT, chemotherapy, and liver-directed treatments. As the field evolves, ongoing clinical trials are playing a transformative role by testing novel agents and combinations tailored to tumor heterogeneity and molecular profiles. Supporting these studies is essential not only to expand therapeutic options but also to improve long-term outcomes and quality of life for patients living with these complex and often indolent malignancies.

Antibody–drug conjugates (ADCs) are emerging as a promising therapeutic strategy for advanced, treatment-refractory neuroendocrine tumors (NETs), offering a targeted approach that could overcome the limitations of conventional therapies. By coupling tumor-specific antibodies with potent cytotoxic payloads, ADCs selectively deliver chemotherapy directly to NET cells, sparing healthy tissues and minimizing systemic toxicity. This precision targeting is particularly valuable in NETs, which often exhibit indolent yet treatment-resistant behavior, leaving patients with limited options after progression on somatostatin analogs, targeted therapies, and radioligand treatments. Early clinical studies are demonstrating encouraging activity, with durable responses even in heavily pretreated populations. As novel ADCs exploiting NET-specific surface antigens advance through development, they hold the potential to redefine the therapeutic landscape and extend survival for patients with advanced disease.

This review focuses primarily on phase II and III studies conducted in the past five years; it also includes selecting early-phase (phase I) trials where relevant. These trials, while limited in sample size and duration, offer critical insight into the future direction of therapy development, particularly in areas such as DNA repair inhibition, targeted radioligands, and bispecific antibodies.

Looking ahead, optimizing patient selection through molecular profiling, leveraging biomarkers like NETest or circulating tumor cells, and fostering multidisciplinary approaches will be essential to tailoring therapy to individual tumor biology. Integrating these diagnostics into treatment decisions could facilitate more effective sequencing, minimize toxicity, and improve outcomes. Continued investment in high-quality clinical trials and global collaboration will be critical to advancing the field and potentially transitioning GEP-NETs from manageable to curable diseases.

## 11. Conclusions

GEP-NETs represent a diverse and biologically complex group of malignancies whose heterogeneity continues to pose diagnostic and therapeutic challenges. Enhancing early detection through sensitive imaging modalities and reliable biomarkers, refining prognostic tools, and tailoring therapy using molecular profiling will be pivotal in achieving more durable disease control. Additionally, optimizing treatment sequencing and evaluating the synergistic potential of combination therapies particularly involving radioligands, immune modulators, and DNA repair inhibitors could shift the treatment paradigm further toward precision oncology. As the field advances, collaborative clinical trials and global research networks will be instrumental in validating novel agents, biomarkers, and therapeutic strategies. The future of GEP-NET management lies in a precision-driven, patient-centered approach that aligns cutting-edge science with comprehensive care moving closer to the goal of improved survival and meaningful quality of life for all patients.
